# Spontaneous Regression of Metastatic Renal Cell Carcinoma after SARS-CoV-2 Infection: A Report of Two Cases

**DOI:** 10.3390/curroncol28050294

**Published:** 2021-09-03

**Authors:** Tomas Buchler, Lukas Fiser, Jaroslava Benesova, Hana Jirickova, Jana Votrubova

**Affiliations:** 1 Department of Oncology, First Faculty of Medicine, Charles University and Thomayer University Hospital, 14059 Prague, Czech Republic; 2 Department of Urology, District Hospital Kladno, 27201 Kladno, Czech Republic; lukas.fiser@nemk.cz; 3 Department of Radiology, District Hospital Kladno, 27201 Kladno, Czech Republic; jaroslava.benesova@nemk.cz; 4 Department of Radiology, Medicon, 14000 Prague, Czech Republic; hana.jirickova@medicon-rdg.cz; 5 Department of Radiology, Thomayer University Hospital, 14059 Prague, Czech Republic; jana.votrubova@ftn.cz

**Keywords:** renal cell carcinoma, spontaneous regression, COVID-19, SARS-CoV-2, innate immunity

## Abstract

Spontaneous regression of metastatic renal cell carcinoma (mRCC) is a rare event, often associated with an activation of innate immunity by various triggers. SARS-CoV-2 infection induces a strong inflammatory response in some patients and a cytokine storm is one of the main causes of severe morbidity and mortality associated with the virus. Here, we describe two cases of patients with histologically and radiologically proven mRCC whose treatment was delayed due to COVID-19 and who experienced spontaneous tumour regression following the infection. Both patients reported here had predominantly pulmonary and mediastinal involvement and underwent nephrectomy. The interval between the diagnosis of COVID-19 and the detection of tumour regression was 3 and 4 months, respectively. Although approved vaccines and other measures are clearly the best way to prevent COVID-19-associated morbidity and mortality in cancer patients, we hypothesize that innate immunity activation by the infection can contribute to tumour regression in special circumstances.

## 1. Introduction

Spontaneous regression of metastatic renal cell carcinoma (mRCC) is a rare but well described phenomenon. It is thought that spontaneous regression may occur in up to 1% of mRCC cases, especially in patients with clear cell histology after the removal of the primary tumour. Spontaneous regressions in mRCC tend to be long-lasting [1,2,3].

Spontaneous regression of cancer has been reported especially in tumours capable of eliciting strong immune responses, such as renal cell carcinoma, melanoma, and B-cell malignancies. A synthetic attempt has identified a concomitant inflammatory condition as a process providing a link between some reports. The aetiology of these inflammatory responses includes not only severe bacterial or viral infections but also vaccinations followed by febrile reactions [4].

Two cases of spontaneous regression of B-cell lymphomas following COVID-19 have been published [5,6], but, to our knowledge, this is the first description of mRCC regression following SARS-CoV-2 infection.

## 2. Case Description

### 2.1. Patient 1

**Figure 1:**
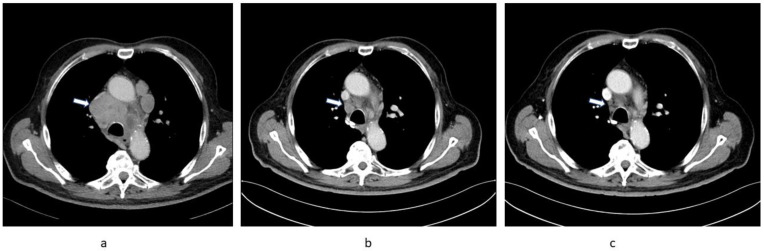
Sequential chest CT images of Patient 1 showing regression of substantial mediastinal lymphadenopathy (arrow). (**a**) 28 August 2020, (**b**) 15 February 2021, (**c**) 18 May 2021.

A 71-year-old male with a medical history of type 2 diabetes and prostatic hyperplasia initially presented with weight loss and exertional dyspnoea in August 2020. A CT scan on 28 August 2020 revealed marked mediastinal lymphadenopathy (Figure 1a) and multiple lung nodules as well as a tumour of the right kidney. A cryobiopsy of an intramural bronchial right lung lesion was reported as a metastasis of grade 2 clear cell renal cell carcinoma (RCC). The patient started to develop signs and symptoms of heart failure confirmed by high levels of N-terminal pro-hormone B-type natriuretic peptide and low left ventricular ejection fraction of 25% at the beginning of October 2020 and was admitted for treatment to an internal medicine department on 10 November 2020. On 21 November 2020, he unexpectedly developed abdominal discomfort and diarrhoea. A PCR test for SARS-CoV-2 was positive. Aside from supportive measures, no specific treatment for COVID-19 was administered. As the cardiac failure had improved by this time, he was discharged after the end of the prescribed quarantine period on 1 December 2020. Right nephron-sparing nephrectomy was planned and carried out on 14 January 2021 confirming the histology of clear cell RCC. The patient was then referred for systemic treatment of mRCC.

At his first oncology appointment he was well, with an Eastern Cooperative Oncology Group (ECOG) performance status of 1. He had a urinary catheter for obstruction due to prostatic hyperplasia. His medication at this time included metformin, spironolactone, furosemide, and metoprolol. Laboratory results were unremarkable except for renal impairment with a glomerular filtration rate (GFR) of 30 mL/min and a moderately increased neutrophil count (8800/µL). A CT scan was ordered to ascertain the extent of the disease prior to planned targeted therapy and was carried out on 15 February 2021; surprisingly, this showed marked regression of all tumour lesions (Figure 1b). Consequently, no treatment was initiated and a follow-up CT scan on 18 May 2021 confirmed ongoing substantial partial regression of metastatic lesions (Figure 1c).

### 2.2. Patient 2

**Figure 2:**
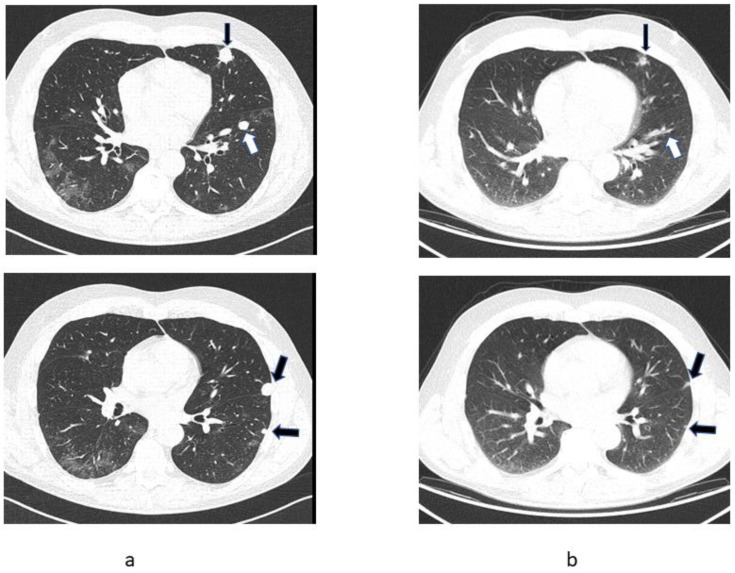
Sequential chest CT images of Patient 2 showing partial regression of pulmonary metastases (arrows). (**a**) CT scan of 15 April 2021, (**b**) CT-registered images from PET/CT scan of 20 July 2021.

A 58-year-old male with no significant medical history presented to his general practitioner on 8 March 2021 with fever and headache. A PCR test for SARS-CoV-2 was positive and the patient was started on antibiotics; firstly, clarithromycin and, subsequently, amoxycillin clavulanate for persistent fever. A chest X-ray on 26 March 2021 showed bilateral lung infiltrates. Therapy with prednisone was initiated and by 14 April 2020 the lung infiltrates had regressed. However, the repeat X-ray also revealed multiple bilateral round dense opacities. The patient was still subfebrile and had lost 10 kg of his initial weight of 105 kg over the previous 8 weeks. A CT scan was ordered and carried out on 15 April 2021 showing post-COVID-19 lung parenchymal changes but also a left renal mass and multiple lung lesions consistent with metastatic cancer (Figure 2a). On 9 June 2021, an uncomplicated left nephrectomy was carried out, with histology reported as poorly differentiated clear cell carcinoma with sarcomatoid features.

At his first oncology appointment on 26 June 2021, the patient was asymptomatic except for mild abdominal discomfort after the nephrectomy. He was taking no regular medication. The physical examination was unremarkable except for mild hypertension. Blood tests showed renal insufficiency with a GFR of 26 mL/min, anaemia (haemoglobin 10.1 mg/dL), mildly increased C-reactive protein (25 mg/L) and increased platelet count (464,000/µL). As a patient with poor prognostic features and sarcomatoid histology, therapy with ipilimumab and nivolumab was contemplated, but a PET/CT was carried out first to assess the current disease extent. The examination on 22 July 2021 showed a marked reduction in lung metastases (Figure 2b). The patient has received no systemic antineoplastic treatment and remains closely followed by an oncologist.

## 3. Discussion

SARS-CoV-2 infection manifests in some patients with a strong inflammatory response with high levels of cytokines such as interleukin (IL)-6 and interferon (INF)-α, and, in severe cases, also IL-2 and tumour necrosis factor [7,8]. The cytokines and chemokines induce T helper 1 (TH1) cell-polarized response attracting monocytes and T lymphocytes to the lungs [8]. An overwhelming cytokine response to SARS-CoV-2 is associated with severe morbidity and mortality. In particular, IL-6 may trigger acute respiratory distress syndrome and is a target for therapeutic intervention with the monoclonal antibody against the IL-6 receptor tocilizumab [9]. The complement system, another component of innate immunity, is also activated in patients with severe COVID-19 infection resulting in a hypercoagulable state [10,11].

Innate immunity has also been used therapeutically in mRCC in the form of the now largely obsolescent cytokine therapy with IL-2 and INF-α. High-dose IL-2, in particular, can induce a durable, complete tumour response in a small number of patients with mRCC [12,13]. Most current immunotherapy protocols emphasize the activation of specific T cells using inhibitors of the programmed cell death protein 1 pathway or the cytotoxic T lymphocyte antigen 4 receptor, producing durable responses in a proportion of patients, even after the treatment is discontinued [14,15]. However, there is an ongoing research effort to mobilize innate immunity to change immunosuppressive tumour microenvironment using indoleamine 2,3-dioxygenase inhibitors, CD40 agonists, and many other approaches [16].

The two patients reported here had predominant pulmonary and mediastinal involvement and, thus, the conditions were favourable for colocalization of the strong innate SARS-CoV-2-induced immune response [8] and specific antitumour immunity. In addition, in both patients, cytoreductive nephrectomy was carried out. The interval between the diagnosis of COVID-19 and the detection of tumour regression was 3 and 4 months in the two patients, respectively. In Patient 1, the response was ongoing after 6 months. There is evidence that both patients had cleared the virus, although we do not know the precise timepoint when this happened. Confirmatory negative tests were not required according to relevant contemporary guidelines. The respective patients tested negative by PCR for SARS-CoV-2 three and six months after the initial detection when testing was carried out in the context of planned medical procedures.

Antibacterial therapy, including fluoroquinolones, may possess certain anticancer, as well as immunomodulatory, activity [17,18]. Clarithromycin may potentiate the effect of tyrosine kinase inhibitors in chronic myeloid leukemia [19]. Anticancer properties of amoxycillin have not been reported, and antibiotic treatment may cause adverse changes in gut microbiota and is associated with the decreased efficacy of cancer immunotherapy [18,20]. Thus, we have no basis to presume that the antimicrobial treatment received by Patient 2 contributed to the tumour regression.

Although in the pandemic environment delays in cancer treatment in general reflect adversely on patient prognosis, in the case of the two reported patients a delay fortuitously allowed us to observe the rare phenomenon. Because spontaneous tumour regression is obviously a rare and unpredictable event, even in the context of the SARS-CoV-2 pandemic, approved vaccines and other preventative measures must be used to prevent morbidity and mortality in cancer patients, including those with potentially immunogenic malignancies. Nevertheless, the question of SARS-CoV-2-induced innate immune response contributing to tumour regression could be retrospectively explored using clinical trial data, where an augmentation of clinical responses after COVID-19 could be potentially detectable, especially in studies investigating immunotherapy.

## 4. Conclusions

Innate immunity activation by SARS-CoV-2 infection can contribute to tumour regression in rare cases.

## Data Availability

The data presented in this study are available on request from the corresponding author.
